# Evaluating somatic tumor mutation detection without matched normal samples

**DOI:** 10.1186/s40246-017-0118-2

**Published:** 2017-09-04

**Authors:** Jamie K. Teer, Yonghong Zhang, Lu Chen, Eric A. Welsh, W. Douglas Cress, Steven A. Eschrich, Anders E. Berglund

**Affiliations:** 1 Department of Biostatistics and Bioinformatics, H. Lee Moffitt Cancer Center and Research Institute, Tampa, FL 33612 USA; 2 Department of Molecular Oncology, H. Lee Moffitt Cancer Center and Research Institute, Tampa, FL 33612 USA

**Keywords:** Somatic mutation, Cancer genomics, Next-generation sequencing, Precision medicine

## Abstract

**Background:**

Observations of recurrent somatic mutations in tumors have led to identification and definition of signaling and other pathways that are important for cancer progression and therapeutic targeting. As tumor cells contain both an individual’s inherited genetic variants and somatic mutations, challenges arise in distinguishing these events in massively parallel sequencing datasets. Typically, both a tumor sample and a “normal” sample from the same individual are sequenced and compared; variants observed only in the tumor are considered to be somatic mutations. However, this approach requires two samples for each individual.

**Results:**

We evaluate a method of detecting somatic mutations in tumor samples for which only a subset of normal samples are available. We describe tuning of the method for detection of mutations in tumors, filtering to remove inherited variants, and comparison of detected mutations to several matched tumor/normal analysis methods. Filtering steps include the use of population variation datasets to remove inherited variants as well a subset of normal samples to remove technical artifacts. We then directly compare mutation detection with tumor-only and tumor-normal approaches using the same sets of samples. Comparisons are performed using an internal targeted gene sequencing dataset (*n* = 3380) as well as whole exome sequencing data from The Cancer Genome Atlas project (*n* = 250). Tumor-only mutation detection shows similar recall (43–60%) but lesser precision (20–21%) to current matched tumor/normal approaches (recall 43–73%, precision 30–82%) when compared to a “gold-standard” tumor/normal approach. The inclusion of a small pool of normal samples improves precision, although many variants are still uniquely detected in the tumor-only analysis.

**Conclusions:**

A detailed method for somatic mutation detection without matched normal samples enables study of larger numbers of tumor samples, as well as tumor samples for which a matched normal is not available. As sensitivity/recall is similar to tumor/normal mutation detection but precision is lower, tumor-only detection is more appropriate for classification of samples based on known mutations. Although matched tumor-normal analysis is preferred due to higher precision, we demonstrate that mutation detection without matched normal samples is possible for certain applications.

**Electronic supplementary material:**

The online version of this article (10.1186/s40246-017-0118-2) contains supplementary material, which is available to authorized users.

## Background

Methods for massively parallel sequencing analysis have matured, even as sequencing technologies have undergone continued, rapid improvement. Detection of somatic mutations in cancer samples has proven to be challenging due to the presence of inherited germline variants, sample heterogeneity, and genomic instability. Somatic mutations can be identified from massively parallel sequencing data by directly comparing the DNA sequence from tumor samples with their matched normal samples. This allows subtraction of the germline variants shared by all cells in an individual, leaving only acquired somatic mutations. Somatic mutations include the important driver mutations that give a cell the growth advantage leading to tumorigenesis [1]. The paired tumor/normal approach to precisely identify somatic mutations has been used in landscape studies that have identified commonly mutated positions and genes in a wide variety of cancers, including recent publications by The Cancer Genome Atlas consortium (for review, see Watson et al. [2]). There are several implementations, including VarScan [3, 4], Shimmer [5], SomaticSniper [6], Strelka [7], and MuTect [8]. However, this approach effectively doubles the number of samples that must be sequenced and analyzed and limits investigation to those tumor samples for which a matched normal tissue sample is available. This approach is theoretically sound and widely used, and many methods have been compared and evaluated, including in the crowdsourced DREAM challenge [9] and others [10]. However, the efficacy of somatic mutation detection without matched normal samples has not been widely studied. Recently, Jones et al. reported that tumor-only mutation detection resulted in large numbers of false positive detections and concluded that tumor-only detection should be used with caution, even though the approach is common in clinical testing [11].

As part of the Total Cancer Care® (TCC) project [12], we have developed a large tumor bank consisting of 26,473 frozen tumor samples. Through collaboration with a pharmaceutical partner, Merck & Co., 3917 samples from 3380 unique individuals were subjected to targeted gene sequencing (TGS) covering 1321 genes of interest. Here, we describe and evaluate a detailed approach for somatic mutation detection without matched normal samples based on a Genome Analysis ToolKit (GATK) [13, 14] pipeline. Although GATK is built on a model assuming a diploid genome that is often not applicable in tumor samples, the tool is widely used for somatic mutation detection. We therefore deliberately choose to evaluate this approach because of the disconnect between theoretical concerns and widespread use. We describe the effect of various filters on mutation detection rates and compare tumor-only mutation detection to paired tumor-normal comparison approaches using TGS data and whole exome sequencing (WES) data from the TCGA project (250 samples from 5 diseases). This study expands on the findings of Jones et al. to allow for a better understanding of the limitations and potential utility of tumor-only mutation detection. We finally describe the detailed pipeline built for this evaluation from publically available analysis tools, allowing researchers to evaluate and utilize tumor-only mutation detection themselves.

## Methods

### Experimental design

The objectives of this study were to define a specific methodology to detect somatic mutations without matched normal samples and to evaluate its performance. As a subset of tumor samples did have a matched normal sample, mutations were also directly compared between tumor-only and tumor-normal mutation detection strategies to calculate precision and recall.

### Study cohorts

**Fig. 1:**
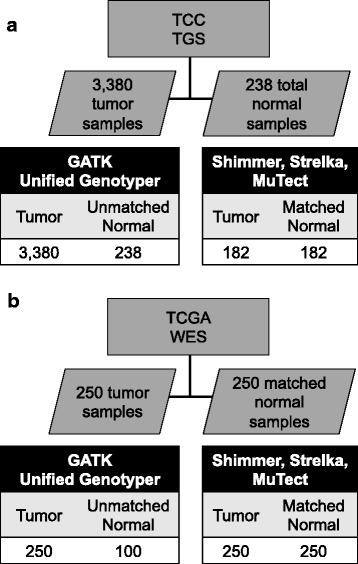
Overview of cohorts. Cohort description and sample counts for the TGS (**a**) and WES (**b**) cohorts

A cohort of samples from 3380 unique individuals (2575 primary solid tumors, 675 metastatic solid tumor, and 130 hematologic malignancies) from the TCC project was utilized in this study (Fig. 1a). Samples were classified according to site of tumor origin (Additional file 1: Table S1). The sites with the highest number of samples include the lung, large bowel, breast, kidney, ovary, and skin. These samples were subjected to TGS across the protein coding exons of 1321 genes covering 3.8 megabases (Additional file 2: Table S2). The median number of reads aligning per sample was 15,283,830. The read depth was consistent across tissue sites of origin with a median depth coverage of 141× (Additional file 3: Figure S1d). The median percentage of targeted bases which covered ≥ 10× across samples was 93.7%. Altogether, 53.4 million reads were generated, for a total of 4.8 trillion bases.

To identify potential batch effects or other confounding factors, a principal component analysis (PCA) was performed on various sequence metrics, including missing values, mutation counts, read counts, and transition/transversion ratios. The majority of samples were grouped together, with two smaller subgroups showing some deviation (Additional file 3: Figure S1a). PCA loadings showed that the 1st component subgroup (tailing out to the right) was affected by missing values and the 2nd component subgroup (tailing to the bottom left) was affected by mutation counts (presumed to be samples with a hyper-mutator phenotype) (Additional file 3: Figure S1b) It was noted that the sites of origin did not cluster together, suggesting that the batch effects were not responsible for similarities between samples within a site of origin.

To assess the extent of potential sample issues using sequencing data and clinical metadata alone, the balance of sequence reads aligning to the X and Y chromosomes was examined to infer gender, which was then matched to clinical data for each patient. The ratio of sequence reads on chromosome X compared to chromosome Y varied over 7 logs, but formed a very distinct bi-modal pattern (Additional file 3: Figure S1c). The high ratio peak was inferred to be female, and empirical cutoffs were set to determine gender across the cohort (male ≤ 4, female ≥ 5.75). Of all samples with determined gender, 0.7% showed a discrepant gender call and were excluded from further analysis.

As a part of the TCC cohort, 238 adjacent normal tissue samples were available from individuals with cancer, which were used to create a normal pool for filtering purposes. Of these, 182 normal samples were paired with a primary tumor sample: matched tumor/normal mutation detection software was applied to these pairs for comparison to tumor-only mutation detection.

WES was also included from a cohort of 250 samples assembled from five different cancers characterized as part of The Cancer Genome Atlas (TCGA) project (Fig. 1b). Fifty tumors each were selected from acute myeloid leukemia (LAML), glioblastoma multiforme (GBM), lung adenocarcinoma (LUAD), ovarian serous cystadenocarcinoma (OV), and skin cutaneous melanoma (SKCM) (Additional file 4: Table S3). These samples each had a matched normal sample, which was used for the matched tumor/normal mutation identification methods. One hundred of these normal samples were selected for a normal pool, which was used for tumor-only mutation detection.

### Tissue samples and consent

Tissue samples were collected according to the TCC methods and consent protocols detailed by Fenstermacher et al. [12].

### TGS sequencing

DNA was subjected to solution-hybridization-selection using SureSelect technology (Agilent Technologies, Santa Clara, CA) targeting 1321 genes. Samples were then sequenced on GAIIx sequencers using a 90 bp, paired-end configuration (Illumina, Inc., San Diego, CA).

### Analysis—alignment

Sequence reads were aligned to the human reference genome (hs37d5) using the Burrows-Wheeler Aligner (BWA) version 0.5.9-r16 [15]. Duplicate reads were marked with Picard-Tools 1.56 (http://broadinstitute.github.io/picard/). Indel realignment and base quality score recalibration were performed with GATK [13, 14]. GenomeAnalysisTKLite-2.2 was used as it was one of the last freely available versions and therefore usable by a wider audience. Samtools v0.1.16 was used for BAM file handling.

### Analysis—tumor-only genotype determination

Multi-sample genotype determination was performed with GATK UnifiedGenotyper. Variant confidence/quality by depth (QD) metric was excluded from Variant Quality Score Recalibration (VQSR), as we have previously found this to penalize variants deviating from the expected 50% alternate allele frequencies. Further modifications to the pipeline are described in Additional file 5. Sequence variants were annotated with ANNOVAR [16], and additional information was included from 1000 Genomes [17] (20110521 release), ESP (http://evs.gs.washington.edu/EVS/, version ESP6500SI-V2), and COSMIC [18] version 61.

### Analysis—matched tumor/normal mutation detection

Tumor-normal mutation detection of SNVs and indels was performed with Shimmer [5] (--minqual 20 –mapqual 16), Strelka [7] (v1.0.13, default settings), and MuTect [8] (v1.1.4, --max_alt_alleles_in_normal_count 3 --max_alt_allele_in_normal_fraction 0.05). In the MuTect analysis, indels were called using GATK SomaticIndelDetector.

### Analysis—secondary

BEDTools [19] was used for overlap calculations. The analysis pipeline, VCF filtering scripts, and other analysis scripts were written in Perl, bash, and R. Principal component analysis was performed using Evince (v2.5.5 (Prediktera AB, Umeå, Sweden)) (http://www.prediktera.se/).

Precision (positive predictive value) was calculated as follows: mutations observed in both test method (GATK tumor-only) and standard method (MuTect) (true positives) divided by total mutations called in test method.

$$ \mathrm{Precision}=\frac{tp}{tp+ fp}=\frac{\mathrm{mutations}\  \mathrm{observed}\  \mathrm{in}\  \mathrm{test}\ \mathrm{AND}\ \mathrm{standard}\  \mathrm{method}\mathrm{s}}{\mathrm{total}\  \mathrm{mutations}\  \mathrm{observed}\  \mathrm{in}\  \mathrm{test}\  \mathrm{method}} $$

Recall (sensitivity) was calculated as follows: mutations observed in both test method and standard method (true positives) divided by total mutations called in standard method.

$$ \mathrm{Recall}=\frac{tp}{tp+ fn}=\frac{\mathrm{mutations}\  \mathrm{observed}\  \mathrm{in}\  \mathrm{test}\ \mathrm{AND}\ \mathrm{standard}\  \mathrm{method}\mathrm{s}}{\mathrm{total}\  \mathrm{mutations}\  \mathrm{observed}\  \mathrm{in}\  \mathrm{standard}\  \mathrm{method}} $$

## Results

### Genotype calling and VQSR tuning

Somatic mutations were identified from tumor samples using BWA [15] alignments and GATK [14] quality improvement and genotype determination. GATK’s UnifiedGenotyper module has the ability to determine the exact genotype of each sample at every variant position when using the multi-sample detection mode. This returns not only a list of variants seen in each sample but also whether non-variant samples have the reference genotype or are “missing” at a position due to insufficient data. This approach is critical to ensure precise classification of samples as mutated or reference for downstream phenotype association analyses. We initially followed the best practices guidelines with several adjustments (see Additional file 5).

BWA-GATK mutation detection was applied to the TGS dataset, and VQSR FILTER status was examined. A high rate of PASS putative mutations was observed, with about 5% of variants being assigned the least specific “SNPto100” tranche. However, with near-default discovery and VQSR settings, only 3.8% of the COSMIC v61 [18] mutations seen more than five times had a value of PASS (Additional file 3: Figure S2b), while 60% were in the least specific tranche (SNPto100). Neither VQSR tuning (removing HaplotypeScore and percentBadVariants) nor adding a cancer mutation training set (COSMIC v61 mutations seen more than once) greatly increased PASS count. The variant list was then filtered on the target regions plus 25 flanking base pairs (total size = 4.9 megabases) before VQSR, resulting in a large increase in the number of final passing COSMIC variants, 86.4%. This also resulted in a greater proportion of all variants passing filter (Additional file 3: Figure S2a). Finally, the target-region pre-filtering was combined with cancer-specific VQSR settings and training, and a COSMIC pass rate of 98.0% was observed. The overall pass rate also increased to 95.5%, suggesting these settings may have reduced specificity (as fewer positions are filtered) but have allowed very high sensitivity for known cancer mutations.

WES samples were less impacted by GATK settings (Additional file 3: Figure S2c, d). The overall PASS rates were slightly higher than in the TGS data, and COSMIC mutations had a much higher PASS rate with default settings (66.3% in WES vs. 3.8% in TGS). No COSMIC mutations were observed in the SNPto100 tranche with any applied settings. Tuned settings applied to the TGS cohort above were also applied to the WES dataset and also increased the proportion of both COSMIC and all PASS mutations. As was observed in the TGS cohort, the highest PASS rate in the WES cohort occurred when the mutations were first target-filtered, and then, VQSR was run using COSMIC training. While less critical, tumor-specific settings benefit whole exome sequencing as well by ensuring known somatic mutations are not falsely removed.

### Efficacy of various filtering strategies

**Fig. 2:**
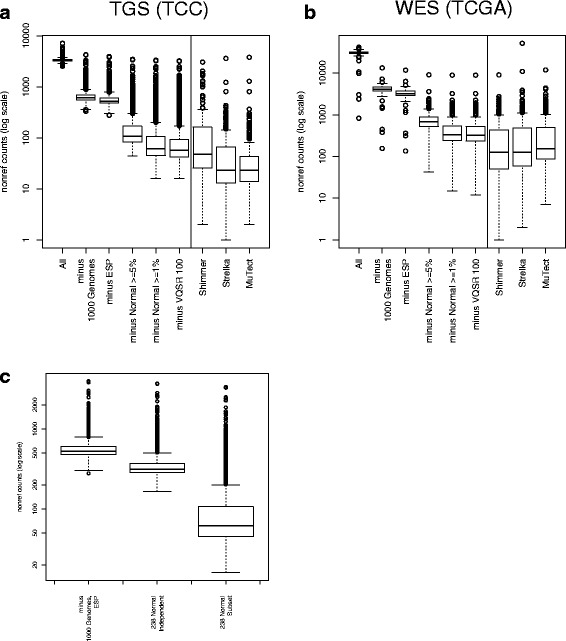
Tumor-only mutation counts with filtering. **a** Boxplot showing numbers of mutations detected in the TGS cohort using tumor-only methods after each filtering step (left) and using matched tumor-normal methods on 182 sample pairs (right). **b** Boxplot showing numbers of mutations detected in the WES cohort using tumor-only methods after each filtering step (left) and using matched tumor-normal methods (right). **c** Boxplot demonstrating that in the TGS cohort, analyzing the normal samples independent of the tumor samples results in reduced ability to remove potential artifacts. GATK variant detection on all tumor and normal samples together, followed by isolation of the normal subset to annotate the tumor samples, results in the removal of more potential artifacts. Median counts are indicated by the dark line in the middle of the box. The bottom and top of the box are the first and third quartiles, respectively. The whiskers represent the most extreme points within 1.5 times the interquartile range. The *y*-axes are in a log scale

Several strategies were applied to enrich for somatic mutations. A median of 3328 potential mutations per sample was detected with the tuned BWA-GATK pipeline (Fig. 2a, “All”). The 1000 Genomes project [17, 20] has cataloged common inherited genetic variation across many different populations around the globe. Variants seen in this dataset were excluded as a first-pass filter for inherited variation, which decreased the putative mutation rate to a median of 608 per sample (Fig. 2a, “minus 1000 Genomes”). The NHLBI Exome Sequencing Project (ESP) dataset, which includes 6503 individuals, was used for further filtering. Although we expected that the large increase in “control” sample numbers would result in the exclusion of many more rare inherited variants, the median mutation count dropped only to 526 (Fig. 2a, “minus ESP”). Several larger population databases have recently become available. We examined the efficacy of filtering with ExAC [21] and KAVIAR [22]. ExAC includes data from > 60,000 whole exomes, although we used the non-TCGA download to avoid filtering known somatic mutations. KAVIAR includes > 13,000 whole genomes and > 64,000 whole exomes (including the ExAC database) and excludes cancer genomes. Although cancer samples should be absent from these databases, we observed common somatic mutations in both, necessitating the use of an allele frequency cutoff. We found that removing variants present in these databases at ≥ 1% allele frequency further decreases the number of detected mutations (Additional file 3: Figure S3a).

Many putative variants are likely artifacts arising from improper sequence alignment [23, 24], and these artifacts tend to be common across samples. We reasoned that variants observed commonly in tumor samples and also in a pool of normal samples are not likely to be cancer driver mutations and are more likely artifacts, especially when common population variants have already been removed. Potential artifacts were identified in a pool of 238 normal samples using the tuned BWA-GATK pipeline. Although these were matched samples, they were treated as unmatched: any mutation observed in this normal pool dataset at greater than or equal to 5% population allele frequency was removed. This dramatically reduced the number of putative tumor mutations to a median of 109 per sample (Fig. 2a, “minus Normal ≥ 5%”). A stricter filter of no more than 1% normal allele frequency further reduced the median putative mutation count to 62 (Fig. 2a, Additional file 3: Figure S3a, “minusNormal ≥ 1%”). Finally, excluding the VQSR Tranche100 variants resulted in a final median mutation count of 57 (“minus VQSR 100”). This resulted in a final median mutation rate of 11.6/megabase investigated.

A subset of the TGS cohort also had matched adjacent normal samples, allowing for a direct comparison of matched tumor/normal mutation methods to our tumor-only pipeline. We applied three different commonly used algorithms for detection of mutations using matched tumor/normal pairs: Shimmer, Strelka, and MuTect. These methods have performed reasonably well in comparisons [9, 10] and are all capable of detecting single nucleotide and deletion/insertion mutations. Somatic mutations were identified in 182 matched primary tumor/normal pairs using Shimmer, Strelka, and MuTect, resulting in a median mutation count of 48.0 per sample (9.83/megabase), 23.0 per sample (4.71/megabase), and 23.0 per sample (4.71/megabase), respectively (Fig. 2a, right-hand side).

The same filtering strategy was applied to the WES cohort tumor-only analysis. As different TCGA Genome Sequencing Centers used different targeted capture designs, the refSeq coding exons (plus 25 flanking base pairs) were used as target regions. Although all 250 tumors had a matched normal sample in the TCGA dataset, a subset of 100 was selected for the normal pool and analyzed together with the tumor samples using the GATK tumor-only mutation detection strategy. Putative mutation counts were determined after each sequentially applied filter. Mutation counts were higher than the TGS cohort due to the larger target size (whole exome vs. 1321 genes). As observed for the TGS cohort, the median putative mutation count decreased with each additional filter (Fig. 2b, Additional file 3: Figure S3b): all detected (31,335), minus 1000 genomes (4099.5), minus ESP (3180.5), minus normal ≥ 5% (680), minus normal ≥ 1% (335), minus Tranche100 (327). A final mutation rate of 7.6/megabase was observed within the refSeq coding target (43.0 megabases, including 25 bp flanking regions). The difference between mutation rates of TGS and WES is most likely due to the differences in cohort makeup (the TGS cohort has more samples from the more highly mutated tumor types). Importantly, the pattern of putative mutation count decrease was similar in the TGS and WES cohorts: large initial decreases when removing 1000 genome variants; modest decreases after further excluding ESP, KAVIAR, and ExAC; and then large decreases after further filtering with a normal pool.

As the WES cohort used TCGA data, matched normal samples were available for all tumors, enabling a direct comparison of tumor-only and matched tumor/normal mutation detection. Similar to the results observed in the TGS cohort, mutation counts were lower with the matched tumor/normal methods (Fig. 2b, right-hand side): Shimmer 125 (2.9/megabase), Strelka 128 (3.0/megabase), and MuTect 154.5 (3.6/megabase).

**Fig. 3:**
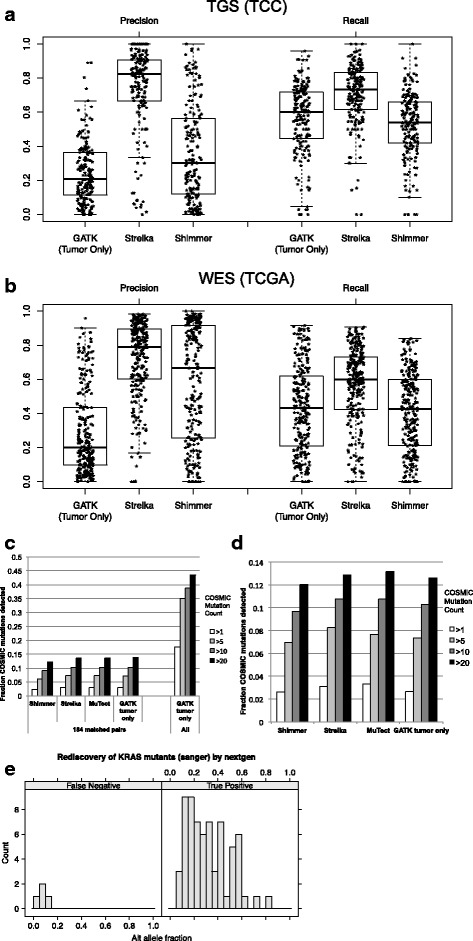
Recall and precision of tumor-only and matched tumor-normal mutation detection. Recall and precision of methods compared to MuTect in TGS (**a**) and WES (**b**). Distributions are represented with box plots, and individual data points are plotted as asterisks. Fraction of COSMIC mutations detected by **c** matched tumor-normal and tumor-only methods within 182 TGS samples (left) and all TGS samples using tumor-only methods (right). **d** 250 WES samples. Shading indicates the number of times the mutation was observed in the COSMIC v61 database. **e** TGS alternate allele fraction and accuracy of *KRAS* G12/G13/Q61 mutations initially discovered by capillary sequencing. Not shown are the seven mutations detected in the TGS but not capillary sequencing

Normal pools proved to be very effective in removing putative variants in both TGS and WES cohorts. Titration experiments were performed to determine filtering effectiveness using fewer normal samples. The resulting mutation counts were compared after filtering with decreasing numbers of normal samples: from 238 down to 12 (TGS cohort) and from 100 down to 20 (WES cohort). Surprisingly, the amount of putative mutations remaining after removing variants with normal sample allele frequency ≥ 1% was stable down to 25 samples (TGS) and 20 samples (WES) (Additional file 3: Figure S4a, b). Furthermore, the exact method of mutation detection in the normal pool affected the results. When the TGS normal samples were analyzed in a separate, independent GATK run, the number of putative artifacts removed was less then when the normal samples were analyzed together with the tumor samples (Fig. 3c). This is likely due to increased prior probability of detection when variants are observed in other samples using GATK multi-sample genotype detection. This suggests it is beneficial to analyze all samples together and then extract the normal samples for frequency calculations.

### Precision, recall, and sensitivity for known tumor mutations

Mutation detection performance was examined by comparing results of each method at each mutated position. MuTect tumor/normal was used as a truth set due to its common usage in TCGA analyses, as well as its reasonable performance in evaluations of tumor/normal methods. Precision and recall were calculated for each sample against MuTect tumor/normal using GATK tumor-only, Strelka tumor/normal, and Shimmer tumor/normal (Fig. 3a, b). GATK tumor-only recall at all positions was similar to Shimmer and slightly decreased compared to Strelka in both TGS and WES datasets. However, the GATK tumor-only precision was much lower than the Strelka in TGS, and much lower than both Strelka and Shimmer in WES data. We also noted precision and recall heterogeneity across samples for each method. Precision and recall were calculated at subsequent filter levels, which demonstrated increasing precision and slightly decreasing recall with each additional filter (Additional file 3: Figures S5 and S6). Although many inherited variants are removed by population filters, precision only moderately increases due to the large number of remaining variants. This suggests it is not possible to precisely identify somatic mutations with current tumor-only methods and that both population filters and a normal pool are important to increase precision.

To further understand the differences between tumor-only and matched tumor-normal mutation detection methods, genotype calls were directly compared at each position. The agreement of calls was counted within each sample, and median values are displayed in Additional file 3: Figure S7. In both TGS and WES cohorts, the tumor-only method shows the most unique mutations. This was more pronounced in the WES dataset, which agrees with the earlier observation of a larger difference in mutation counts between tumor-only and tumor-normal methods (Fig. 2). Interestingly, Shimmer called many more unique mutations than other tumor-normal methods in the TGS dataset, but MuTect called many more unique mutations in the WES cohort. Positions at which all methods made the same mutation call were the more common (~ 5-fold) than any other combination of two or more methods. Despite this agreement, we noticed that many positions were called by some tumor/normal methods and not others (Additional file 3: Figure S8). Strelka had the highest degree of overlap with the other two methods in both cohorts.

Sensitivity for known cancer mutations was assessed by determining the fraction of COSMIC mutations observed in each dataset. 43.5% of those positions observed commonly in COSMIC (more than 20 times) were also observed in the tumor-only TGS analysis (Fig. 3c, right). This fraction decreased as the less common mutations were considered, likely due to the increased numbers of the more rare mutations as well as false positives (artifacts and common variants) in COSMIC. The fraction of COSMIC mutations detected in 182 TGS tumor/normal pairs was calculated from three different tumor-normal pair mutation detection methods (Shimmer, Strelka, MuTect) as well as from the GATK tumor-only method. The fraction of COSMIC bases observed was highly similar in all methods (Fig. 3c, left). Similar results were observed in the WES cohort: the tumor-only mutation detection method had similar sensitivity as the three different tumor-normal methods (Fig. 3d). This suggests the tumor-only approach has equivalent sensitivity to detect known cancer mutations.

Sample heterogeneity presents a specific challenge for sensitive detection of somatic mutations. The fraction of reads with a mutated base can be lower than expected due to normal sample contamination, mutational heterogeneity within the tumor, or chromosomal amplification. We therefore examined the sensitivity of our final tuned pipeline to detect low-frequency mutations. One hundred ninety of the lung samples that underwent TGS were also subjected to capillary sequencing at the G12/G13/Q61 *KRAS* loci. The capillary sequence data were analyzed with both automated genotype calling pipelines and extensive manual review of chromatograms in order to detect low-frequency mutations. Of the 70 mutations detected by capillary sequencing, 66 were also detected using the tumor-only method. Of the four mutations not detected, three had evidence of the variant, with allele frequencies less than 10% (Fig. 3e). Therefore, given the median overall target coverage of 141×, we observed a mutation allele frequency sensitivity limit of ~ 10%. In addition, seven mutations were detected only in TGS data (not in capillary), with allele frequencies ranging from 8 to 29% (mean = 18.0%, median = 15.6%).

### Pan-cancer somatic mutation rates

**Fig. 4:**
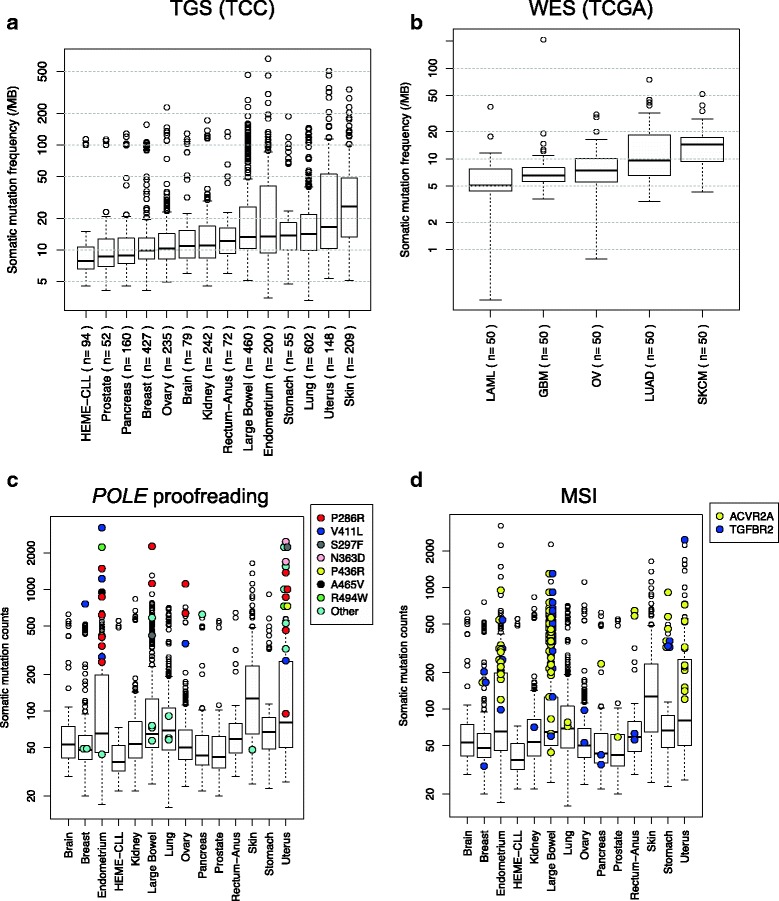
Somatic mutation rates across different tissue types using the tumor-only method. Boxplot of mutation rates for tissue sites of origin in **a** TGS (sites with more than 50 samples, a total of 3035 samples) and **b** WES. **c** The colored dots identify the samples with the indicated *POLE* exonuclease domain mutation. **d** Homopolymer run mutations (the presence of *ACVR2A* and *TGFBR2* mutations side by side infers MSI status). The *y*-axes are in a log scale

Mutation rates detected by our tumor-only pipeline were calculated across different tumor sites of origin in TGS (sites with ≥ 50 samples) and WES cohorts. The frequencies observed using tumor-only mutation detection were higher than those from a matched tumor/normal large consortium sequencing project [25] (Fig. 4a, b). We observed a high variability in mutation rates in skin, uterus/endometrium, lung, and large bowel in agreement with the previous large consortium studies. Skin, uterus, and lung have the highest mutation rates in the “tumor-only” analyses; LAML and CLL have the lowest, mirroring other studies. This suggests that somatic mutations can be greatly enriched using tumor-only methods, but not as precisely identified as matched tumor/normal methods. Our observations have reproduced the overall mutation frequency patterns of large global consortium studies in a completely independent cohort.

### Molecular associations with high mutation burden

Although the median mutation rate varies across tumor sites, every site has outlier tumors with very high mutation rates. To identify the potential molecular causes of high mutation rates, we examined DNA polymerase epsilon (*POLE*) exonuclease domain mutations (amino acids 268-471 and R494 in NP_006222). The recurrent *POLE* mutations almost always occur in the highly mutated outlier samples (Fig. 4c). Singleton *POLE* mutations fell into two groups: highly and moderately mutated tumors. Highly mutated samples with *POLE* mutations were primarily observed in tumors originating from the endometrium/uterus, large bowel, and ovary. One highly mutated sample with a *POLE* mutation was observed in breast and pancreatic cancers; the *POLE*-mutated sample was the most highly mutated in each site.

We have recently observed that the presence of homopolymer indels in both *TGFBR2* and *ACVR2A* is highly correlated with microsatellite instability (MSI) in TGS colorectal samples [26]. Here, we demonstrate that these samples have high mutation rates in the large bowel (Fig. 4d), strengthening the link between *ACVR2A+TGFBR2* mutation and MSI status. Additionally, several putative MSI samples in stomach (2), endometrium (3), and breast (1) tumors were observed. Notably, *ACVR2A*+*TGFBR2* mutations and *POLE* mutations only co-occurred once (singleton *POLE* mutation). These observations confirm and extend previously observed correlations of specific mutations and elevated mutation rates. Many highly mutated samples remained unexplained and require further analysis to identify mutational mechanisms.

## Discussion

**Fig. 5:**
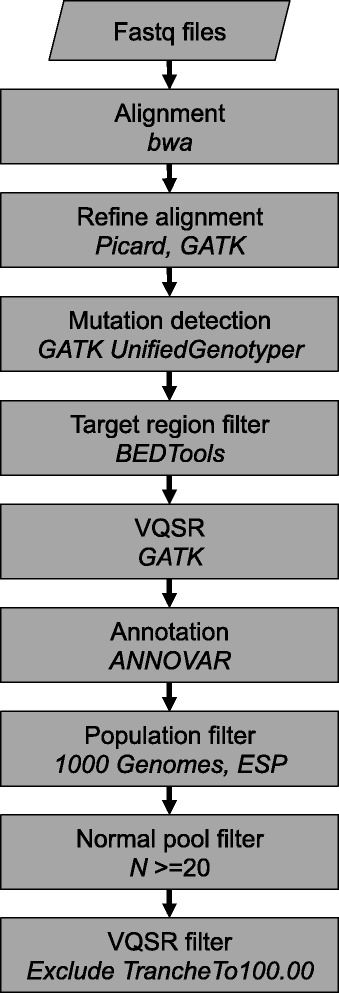
Schematic of tumor-only mutation calling pipeline. Analytical pipeline overview for tumor mutation calling with a subset of matched normal samples. See Additional file 5 for details of commands, options, and settings

We have presented an analysis strategy for the detection of somatic mutations from tumor samples without matched normal samples (Fig. 5, Additional file 5). GATK modifications for detecting somatic mutations included (1) limiting putative mutations to targeted regions, (2) using a VQSR training set of known cancer mutations, and (3) tuning of settings (see Additional file 5) These modifications had a greater impact on reducing false negatives in smaller target sets. Tuning GATK also improved the filter quality of known mutations in whole exome data, although fewer false negatives were initially observed. The use of population genomic datasets (1000 Genomes) was effective at removing the vast majority of likely inherited variants, although the large number of remaining false positive mutations resulted in a small increase in precision. Interestingly, the addition of larger population-specific germline datasets (via the Exome Sequencing Project, ExAC, and KAVIAR) each only removed a modest fraction of additional common variants compared to decreases observed after filtering with a normal pool. Indeed, filtering with ExAC and KAVIAR after the normal pool resulted in removal of only a few variants (Additional file 3: Figure S3). While population databases are helpful in removing inherited variants, they often include overlapping samples and can contain known somatic mutations even when cancer samples have been removed. Therefore, investigators should review population datasets carefully before using them as a filter.

We demonstrated and quantitated the utility of excluding variants observed in a subset pool of normal samples to enrich for somatic mutations. Arbitrary normal pool variant frequency cutoffs of ≥ 5 and ≥ 1% were selected to ensure that known cancer mutations were not erroneously removed. Setting a normal pool allele frequency threshold is important: we observed common cancer mutations (*BRAF* V600E, *PTEN* truncation, *TP53* G245C) in a single normal sample each (TGS cohort). The TGS normal samples were the adjacent normal tissue from surgical resections, which may have contained infiltrating tumor cells. This risk is lessened with peripheral blood normal samples, but observations of circulating tumor cells or cell-free tumor DNA (reviewed in [27]) suggest that a normal pool allele frequency cutoff should be used to avoid false negatives. We also recommend examining the normal sample variants for any well-known cancer mutations to avoid erroneous exclusion. Although we describe mutation detection in tumors without matched normal samples as “tumor-only,” the pool of normal samples is very important for reducing false positive variants and should be included in experimental design.

We observed a relatively small difference in the number of putative mutations removed using differently sized normal sample pools. Indeed, as few as 20 to 25 normal samples may be used to effectively remove many potential artifacts from a large tumor sequencing dataset. We also found that it was important to identify variants using GATK multi-sample genotype detection on the combined set of tumor and normal samples. Our final results show that more putative mutations were detected compared to that in three tumor/normal comparison methods. When compared to MuTect, a commonly used tumor/normal method, GATK tumor-only mutation detection, showed similar sensitivity/recall to tumor/normal methods. However, precision was much lower. Many of these “mutations” are likely to be very rare inherited variants that could only be removed by direct tumor/normal comparison, although future method improvements and larger population variant databases may improve precision. Interestingly, the overlap of calls among precise tumor/normal mutation detection was also imperfect, highlighting the challenge of somatic mutation detection in heterogeneous tumor samples. Although numerous filters were applied to increase mutation specificity, tumor-only sensitivity to detect known mutations was equivalent to tumor/normal pair mutation detection. Comparison with manually reviewed capillary sequencing data demonstrated high tumor-only sensitivity (down to 10% mutant allele frequency) in heterogeneous tumor samples. Therefore, tumor-only sequencing supplemented with a normal pool is an effective alternative to paired tumor/normal sequencing, with similar recall but reduced precision.

Recently, Jones et al. [11] described tumor-only sequencing in 58 targeted samples (111 genes) and 100 whole exomes. We have extended their observations by examining 3380 TGS samples (1321 genes) and 250 WES samples (drawn from 5 diseases included in TCGA). Our observations of high sensitivity for known mutations, but lower precision (especially in WES), agree with those of Jones et al.. We have additionally demonstrated significant non-overlap between three different matched tumor/normal methods. This suggests differences between tumor-only and tumor/normal methods may be due to inaccuracies in both analysis strategies. While Jones et al. conclude tumor-only methods may not be sufficient for high accuracy in the clinical setting, we find the high degree of sensitivity may be useful for tumor classification. Finally, we offer detailed methods to enable others to utilize tumor-only mutation detection.

The observed recall and precision of the tumor-only approach with a limited normal pool suggests that this method lends itself to specific use cases. It has been suggested [25] that there are still many cancer genes and mutations to be discovered, requiring sequencing of many more samples. Although the tumor-only approach results in almost half the cost to reach a target sample size (as each tumor no longer needs a matched normal sample), our analysis demonstrated much lower precision that would result in many more false positive mutations. We conclude that tumor-only approaches are not appropriate for novel mutation detection. However, given similar sensitivity to detect known cancer mutations in heterogeneous tumor samples with greatly reduced cost, the tumor-only detection method can be useful for characterization of known mutations. Indeed, we reproduced earlier observations of mutation rate patterns across diseases. We also identified mutations in the exonuclease domain of *POLE* and mutations associated with MSI as two mechanisms explaining highly mutated samples. MSI mutations were observed in large bowel but also occasionally in endometrium and stomach tumors. *POLE* mutations were observed in many tumor types: endometrium/uterus, large bowel, ovary, pancreas, and breast. Sample classification by known recurrent mutations enables the critical next step in cancer genomics: association of genomic alterations with clinical phenotype. The almost doubling of sample size at a given cost gained from omitting matched normal samples can dramatically improve power to associate mutations with phenotype, but researchers must consider the loss of precision that results when matched normal samples are not used.

## Conclusions

Detection of somatic mutations using tumor samples and a smaller subset of normal samples is a valid strategy for cancer genomics studies. The use of population datasets (1000 Genomes) reduces the number of inherited variants and artifacts. However, filtering against a pool of normal samples captured and sequenced with the same technology increases precision noticeably and should be considered a required part of a “tumor-only” sequencing experiment. We find that recall across all observed mutations is similar to matched tumor/normal methods, and sensitivity for known cancer mutations is equivalent. However, the precision is lower: many putative mutations detected by the tumor-only method are not observed in matched tumor/normal methods. Therefore, tumor-only detection methods as described here are appropriate for characterization of known mutations in samples. Matched tumor/normal mutation detection is more appropriate for applications requiring high precision such as novel mutation detection and mutation signature analysis and remains the optimal approach, especially as sequencing costs continue to decrease.

## Additional files

Additional file 1: Table S1. Sample counts by the site of tumor origin. (XLSX 35 kb)

Additional file 2: Table S2. List of 1321 targeted genes. (XLS 83 kb)

Additional file 3: Figure S1. Large tumor dataset quality control metrics. A. Principal component analysis and B. loadings using sequencing metrics. Colors in A. represent the different tissue sites of origin. C. Ratio of sequence reads aligning to the X and Y chromosome and cutoffs used to infer gender. D. Histogram of average coverage over targeted bases (filtered, aligned reads). **Figure S2:** VQSR filtering effects on tumor-only mutation detection. A. Fraction of total putative TGS mutations falling in each GATK VQSR tranche (PASS being the most specific, SNPto100 being the least specific). B. Fraction of TGS mutations seen in COSMIC more than five times falling into each VQSR tranche. C. Fraction of total putative WES mutations falling in each GATK VQSR tranche (PASS being most specific, SNPto100 being least specific). D. Fraction of WES mutations seen in COSMIC more than five times falling into each VQSR tranche. **Figure S3:** Mutation counts after filtering with additional population databases. Boxplots showing numbers of mutations detected after filtering with KAVIAR, ExAC, or both (excluding AF ≥ 1%) in addition to 1000 Genomes and ESP. The rightmost columns show the minimal effect of filtering with KAVIAR and ExAC after the normal filter has been applied. A. TGS cohort, B. WES cohort. Median counts are indicated by the dark line in the middle of the box. The bottom and top of the box are the first and third quartiles, respectively. The whiskers represent the most extreme points within 1.5 times the interquartile range. The *y*-axes are in the log scale.** Figure S4: **Normal pool features affect the ability to remove variants. Boxplots showing the putative mutation counts after filtering with titrated sample counts in the normal pool for A. TGS cohort, B. WES cohort.** Figure S5: **Total nonref counts, precision, and recall with subsequent filters. Total nonref counts (left), precision compare to MuTect (middle), and recall compared to MuTect (right) for A. TGS and B. WES. All plots are in a linear scale.** Figure S6:** Precision-recall curve. Plot showing approximate precision vs recall for A. TGS and B. WES. Data point circles are area-proportional to the number of putative mutations at each filter level. Note the largest circle across the middle of the plots corresponds to precision = 0, recall = 1. Also note that data point circle sizes are scaled to fit, and the scaling factors are different for TGS and WES. The red line indicates performance of the random classifier based on positives (median number of MuTect call)/total positions (targeted bases). **Figure S7:** Overlap between mutation calls across four methods. Non-area-proportional Venn diagram showing median mutation counts across samples called by each combination of methods. The bold underlined values are the intersection of all the four methods. The underlined values are the counts unique to each method. A. TGS (TCC) cohort and B. WES (TCGA) cohort. **Figure S8:** Overlap between mutation calls across three matched tumor/normal methods. Non-area-proportional Venn diagram showing median mutation counts across samples called by each combination of methods. The bold underlined values are the intersection of all the three methods. The underlined values are the counts unique to each method. A. TGS (TCC) cohort and B. WES (TCGA) cohort. (PDF 945 kb)

Additional file 4: Table S3. List of TCGA samples used. (XLSX 63 kb)

Additional file 5: Methods: A detailed description of the analysis methods used for the detection of somatic mutations with an unmatched pool of normal samples. (DOCX 106 kb)
