# Supplementary material for: Evaluating somatic tumor mutation detection without matched normal samples
Source: Hum Genomics. 2017 Sep 4;11:22. doi: 10.1186/s40246-017-0118-2 (PMC5584341; doi:10.1186/s40246-017-0118-2)

Figure S1

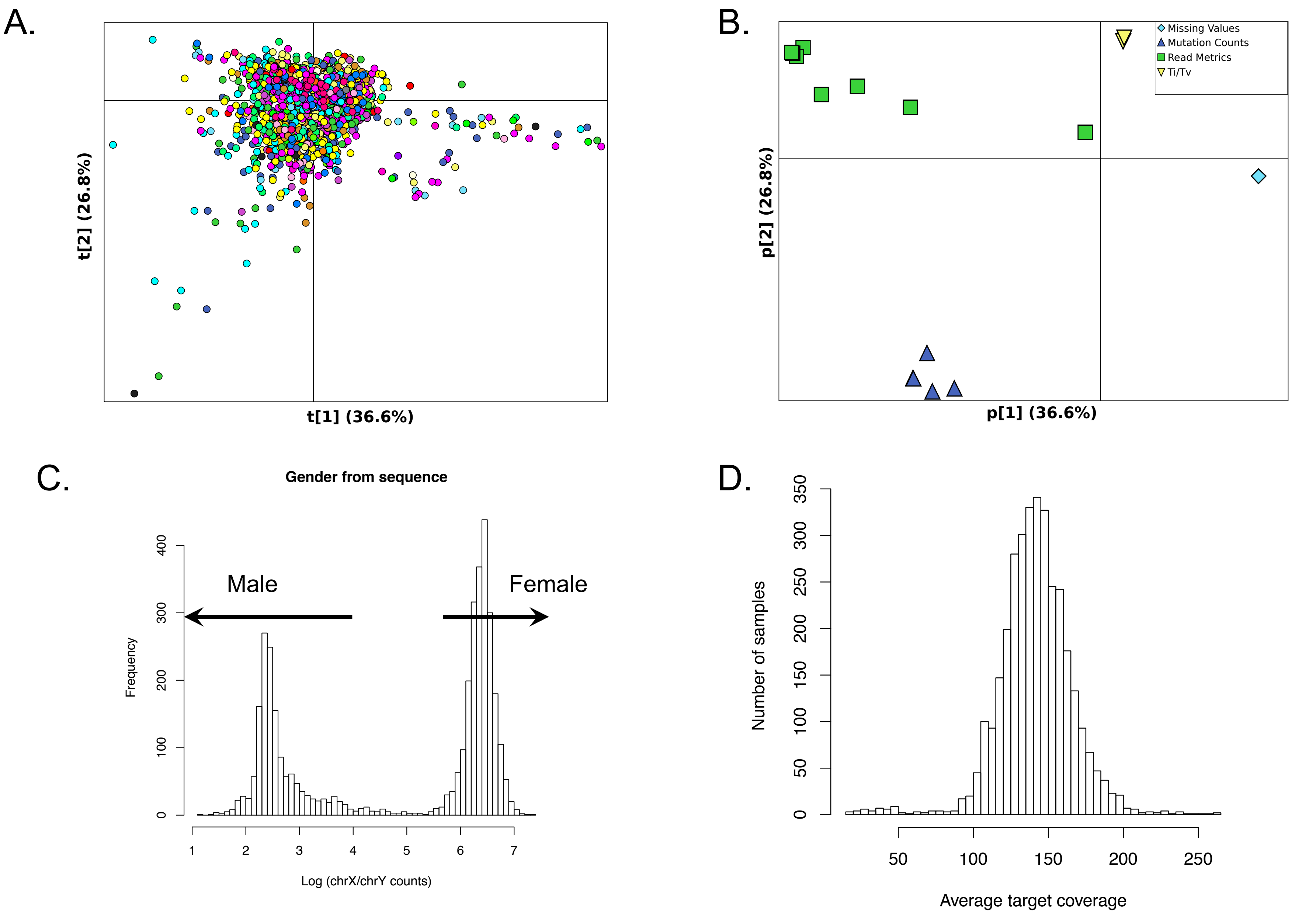

A.
t[2] (26.8%)
t[1] (36.6%)
B.
Missing Values
Mutation Counts
Read Metrics
Ti/Tv
p[2] (26.8%)
p[1] (36.6%)
C.
Gender from sequence
Male
Female
Frequency
0
100
200
300
400
1
2
3
4
5
6
7
Log (chrX/chrY counts)
D.
Number of samples
0
50
100
150
200
250
300
350
50
100
150
200
250
Average target coverage

Figure S2

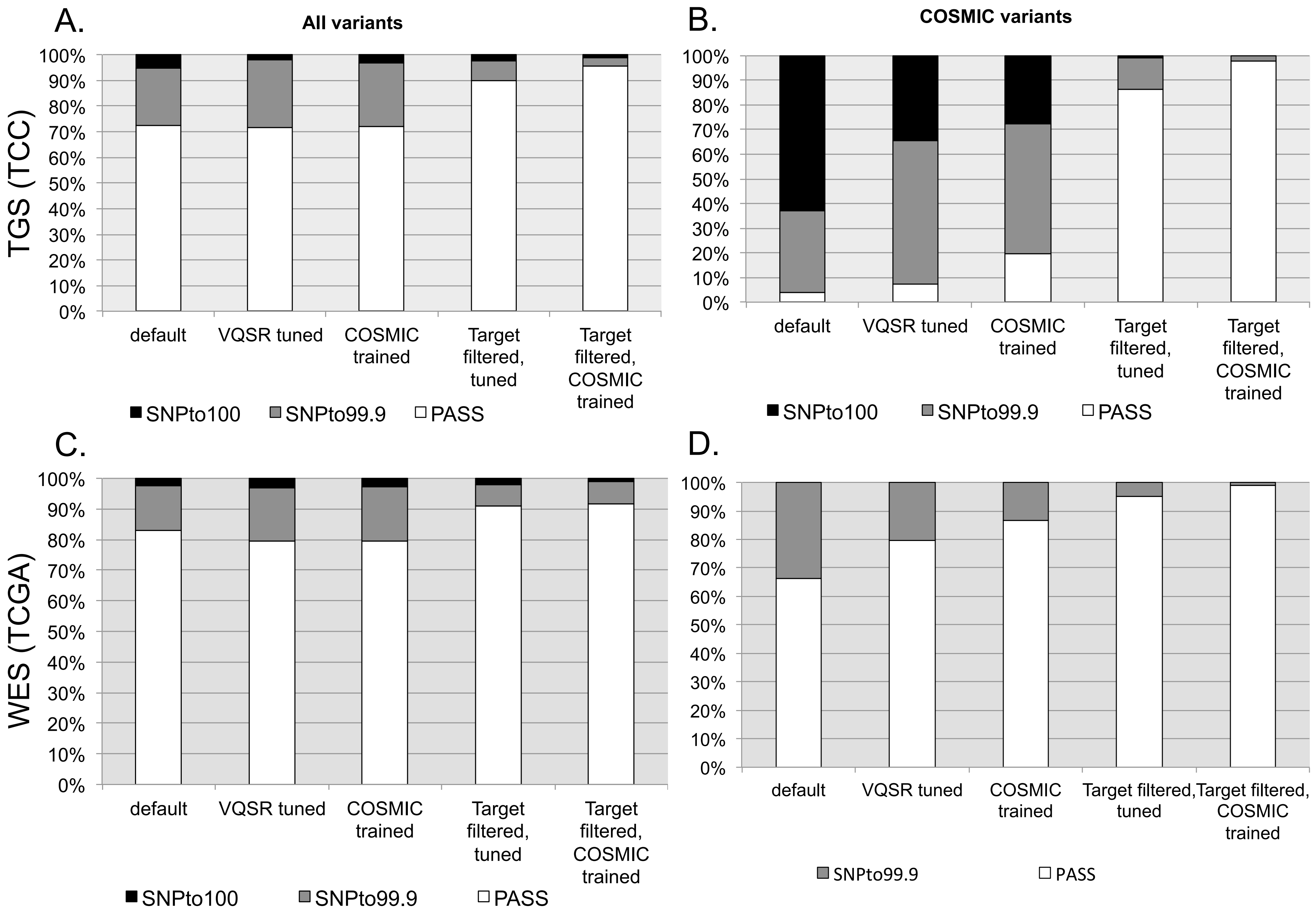
A.
All variants
TGS (TCC)
100%
90%
80%
70%
60%
50%
40%
30%
20%
10%
0%
default
VQSR tuned
COSMIC trained
Target filtered, tuned
Target filtered, COSMIC trained
SNPto100
SNPto99.9
PASS
B.
COSMIC variants
default
VQSR tuned
COSMIC trained
Target filtered, tuned
Target filtered, COSMIC trained
SNPto100
SNPto99.9
PASS
C.
WES (TCGA)
default
VQSR tuned
COSMIC trained
Target filtered, tuned
Target filtered, COSMIC trained
SNPto100
SNPto99.9
PASS
D.
default
VQSR tuned
COSMIC trained
Target filtered, tuned
Target filtered, COSMIC trained
SNPto99.9
PASS

Figure S3

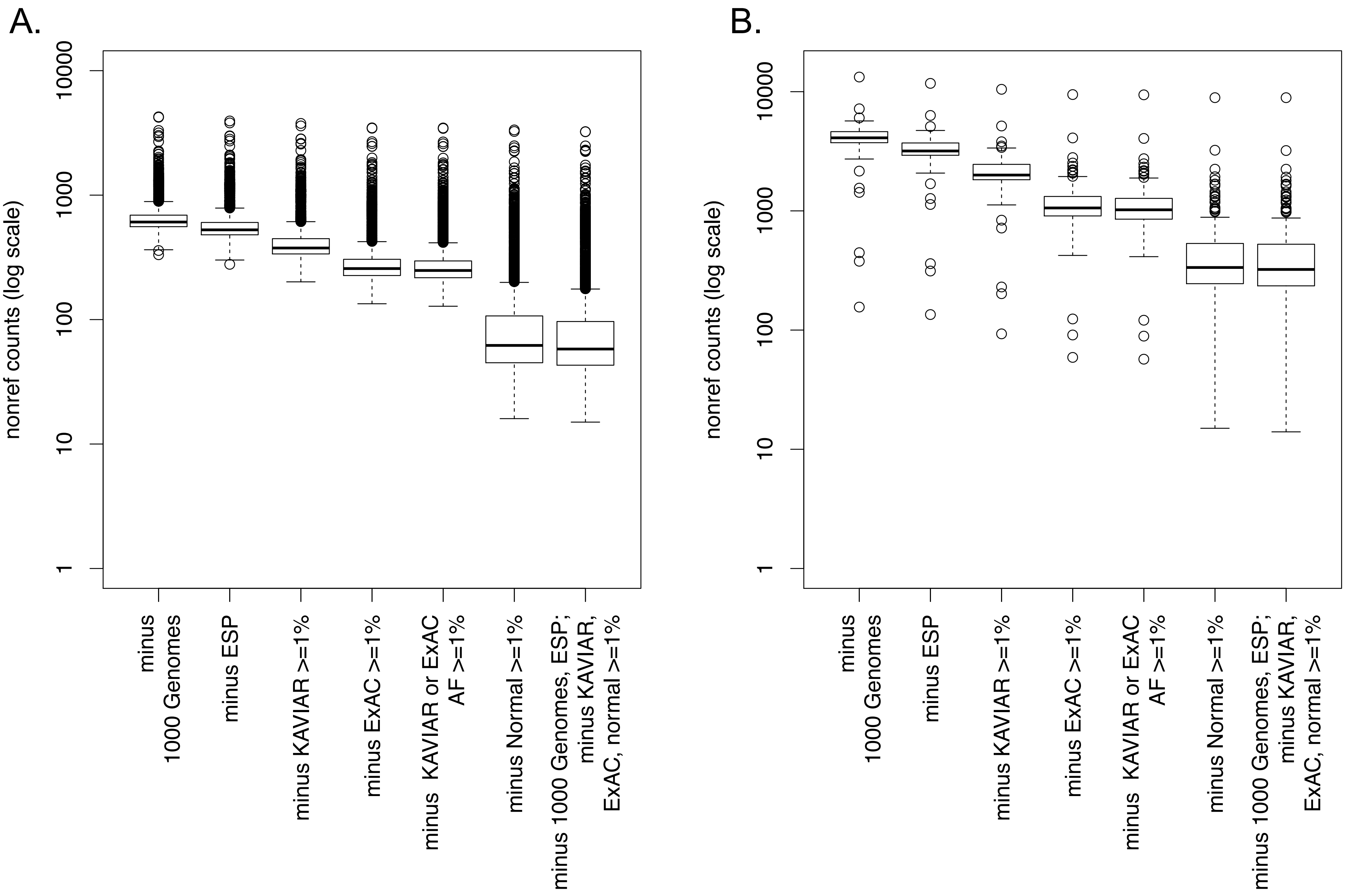
A.
B.
nonref counts (log scale)
10000
1000
100
10
1
minus 1000 Genomes
minus ESP
minus KAVIAR >=1%
minus ExAC >=1%
minus KAVIAR or ExAC AF >=1%
minus Normal >=1%
minus 1000 Genomes, ESP; minus KAVIAR, ExAC, normal >=1%

Figure S4

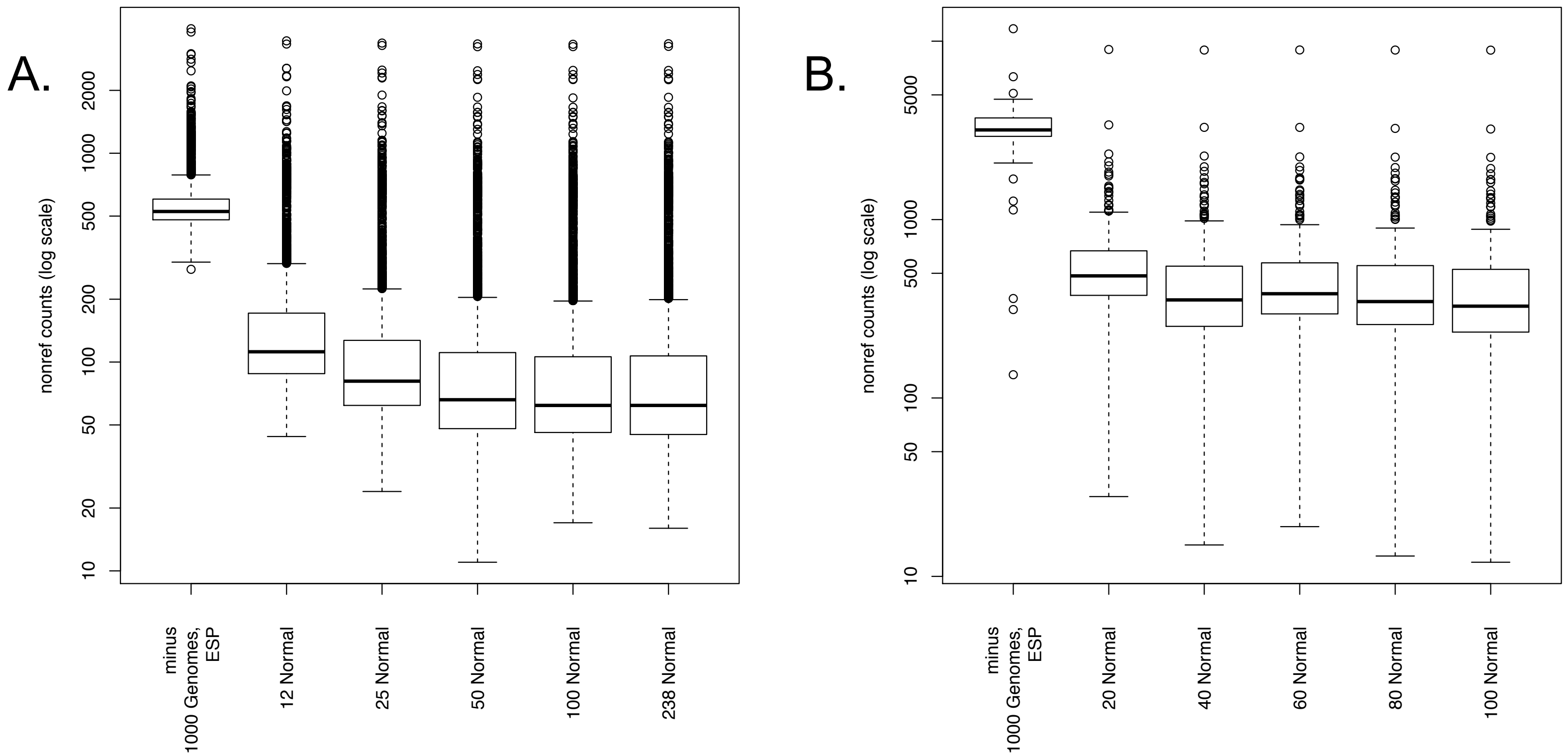

A.
nonref counts (log scale)
10
20
50
100
200
500
1000
2000
minus 1000 Genomes, ESP
12 Normal
25 Normal
50 Normal
100 Normal
238 Normal
B.
nonref counts (log scale)
10
50
100
500
1000
5000
minus 1000 Genomes, ESP
20 Normal
40 Normal
60 Normal
80 Normal
100 Normal

Figure S5

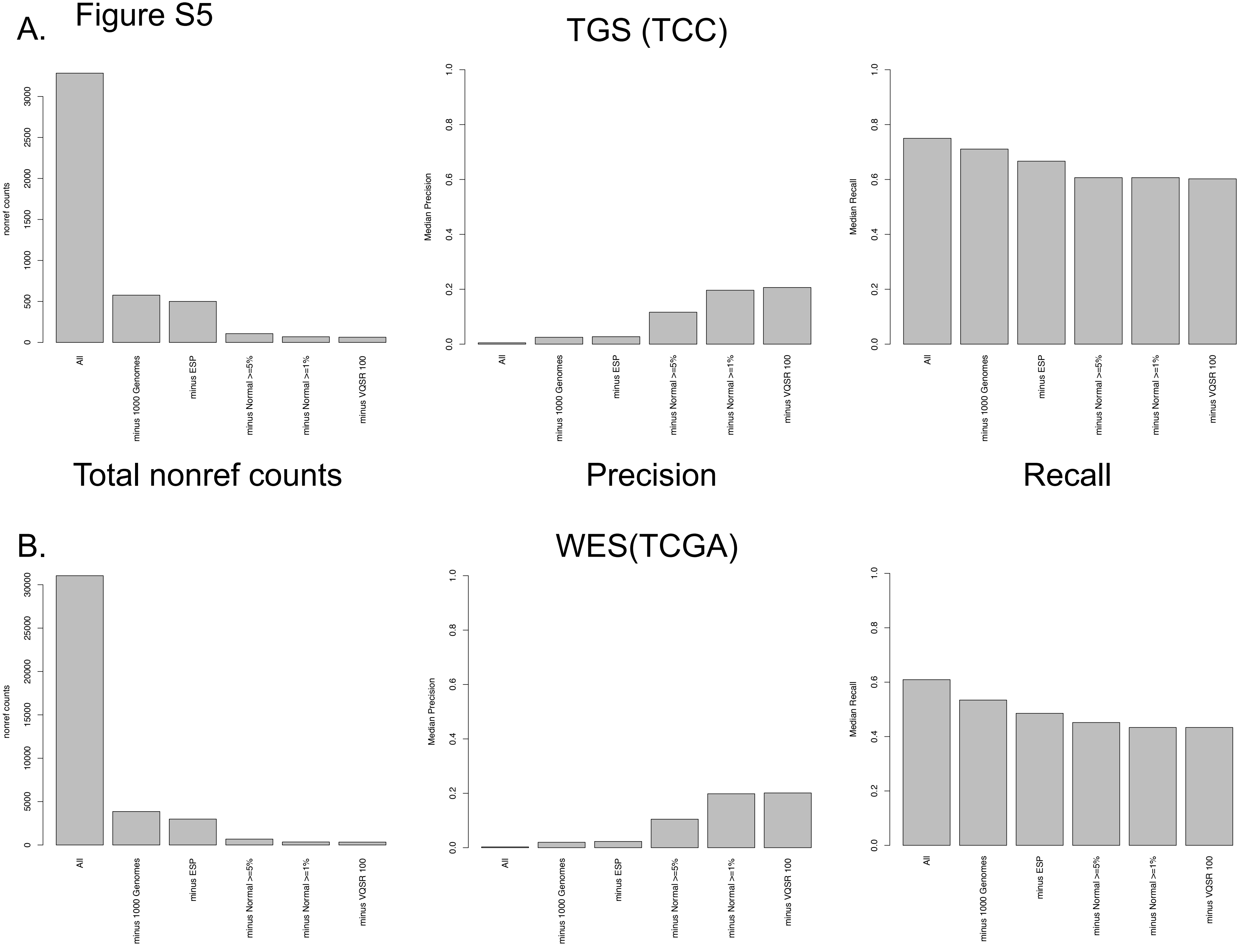
A.
TGS (TCC)
nonref counts
Median Precision
Median Recall
All
minus 1000 Genomes
minus ESP
minus Normal >=5%
minus Normal >=1%
minus VQSR 100
Total nonref counts
Precision
Recall
B.
WES(TCGA)
nonref counts
Median Precision
Median Recall
All
minus 1000 Genomes
minus ESP
minus Normal >=5%
minus Normal >=1%
minus VQSR 100

Figure S6

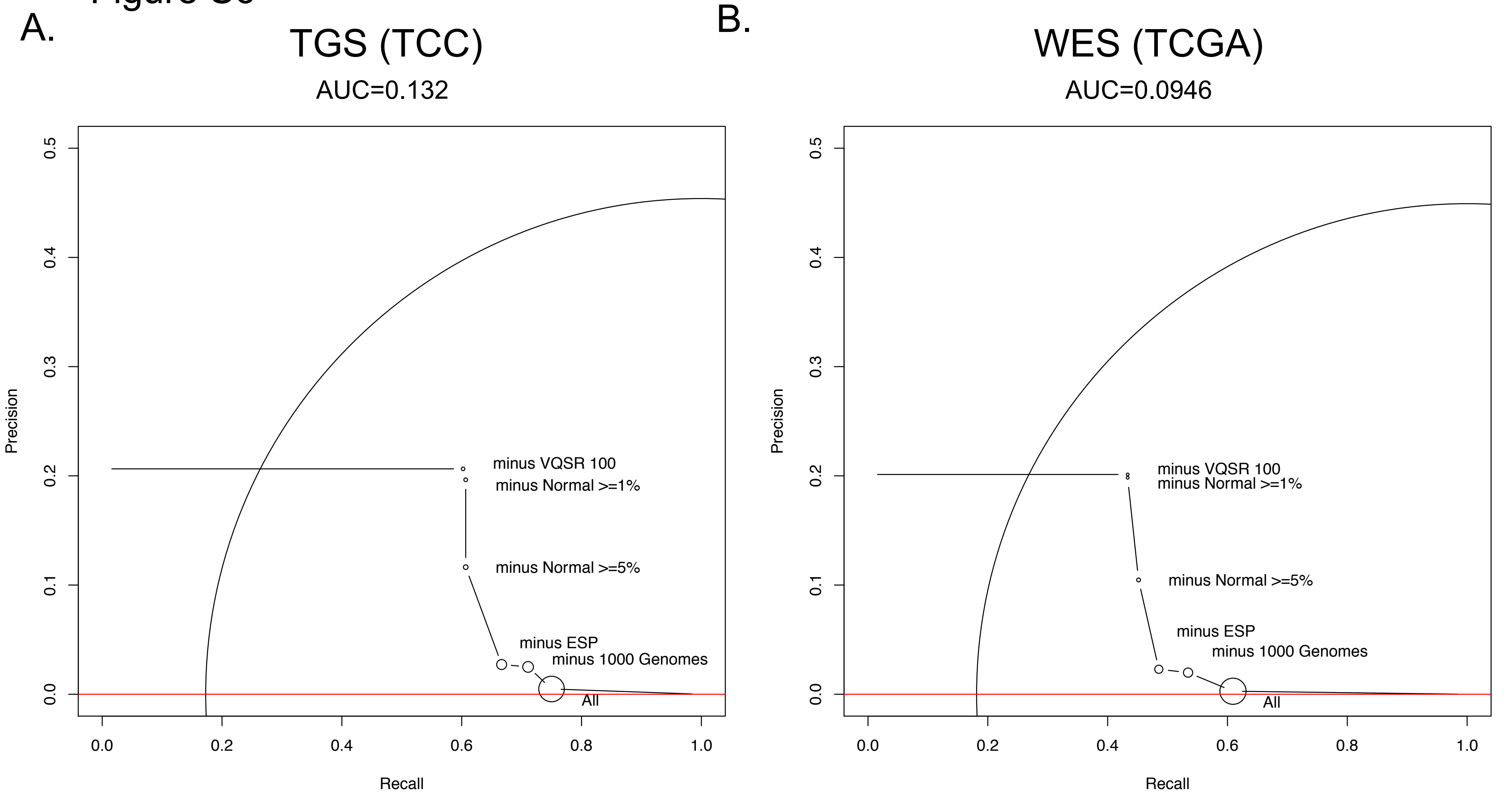
A.
TGS (TCC)
AUC=0.132
Precision
Recall
0.0
0.1
0.2
0.3
0.4
0.5
0.0
0.2
0.4
0.6
0.8
1.0
minus VQSR 100
minus Normal >=1%
minus Normal >=5%
minus ESP
minus 1000 Genomes
All
B.
WES (TCGA)
AUC=0.0946
Precision
Recall
minus VQSR 100
minus Normal >=1%
minus Normal >=5%
minus ESP
minus 1000 Genomes
All

Figure S7

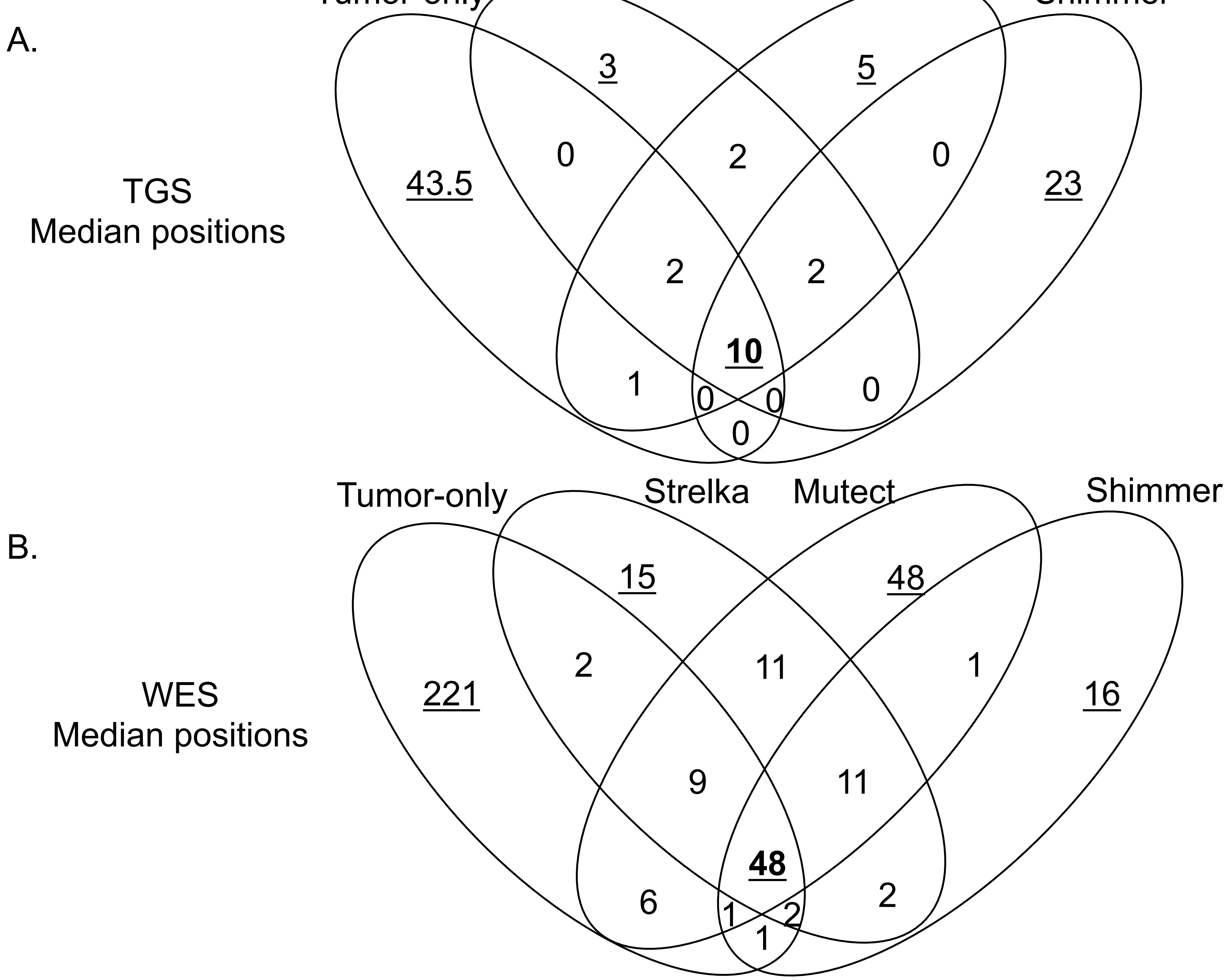
A.
Tumor-only
Strelka
Mutect
Shimmer
TGS
Median positions
3
5
0
2
0
43.5
23
2
2
10
1
0
0
0
0
B.
Tumor-only
Strelka
Mutect
Shimmer
WES
Median positions
15
48
2
11
1
221
16
9
11
48
6
1
2
2
1

Figure S8

A.

TGS, matched Tumor-Normal
Median positions

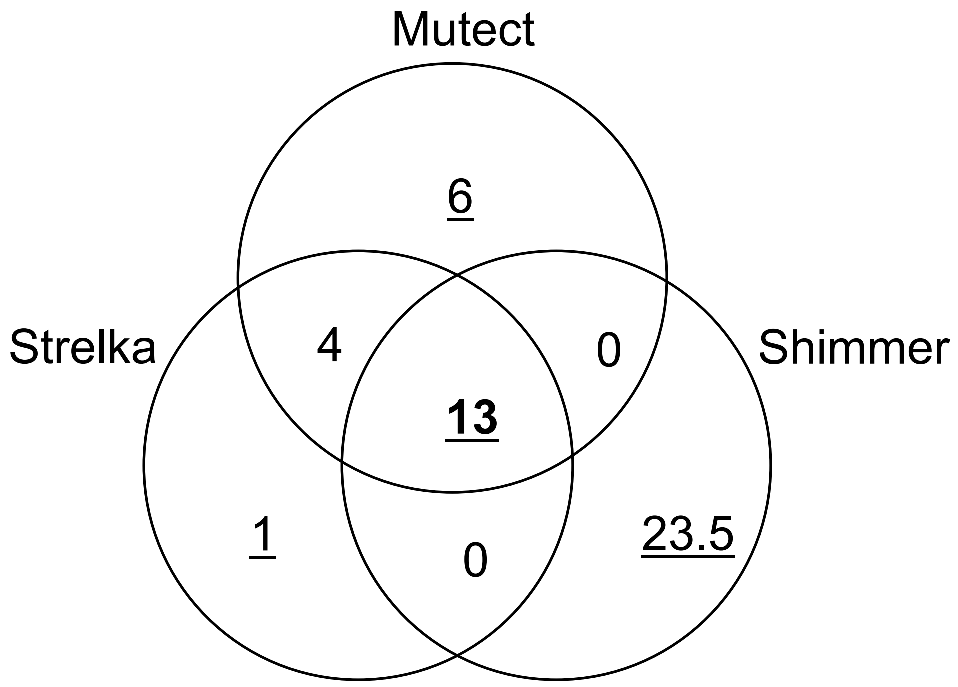

B.

WES, matched Tumor-Normal
Median positions

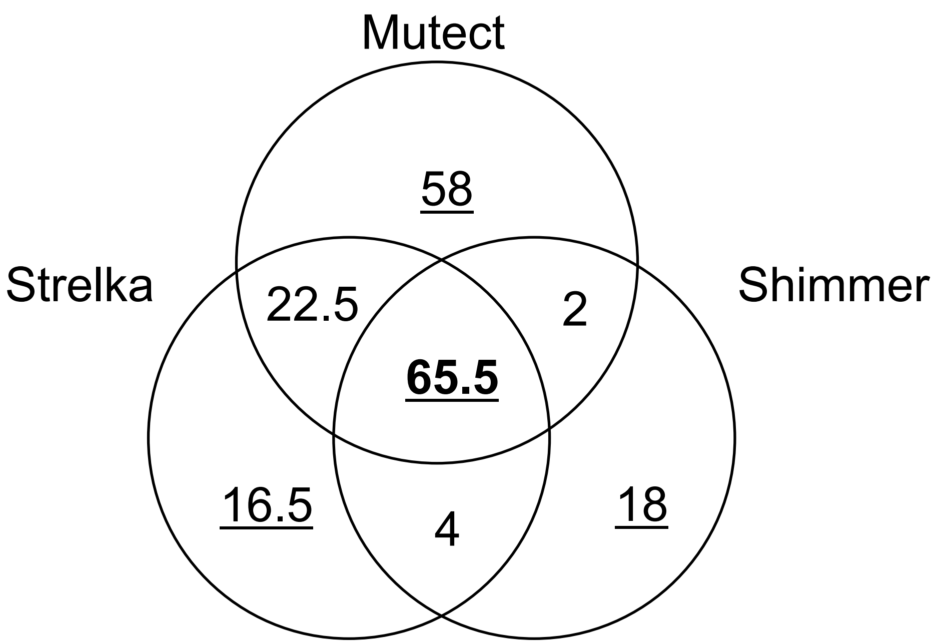

Supplement: Supplementary file 3 — Large tumor dataset quality control metrics. A. Principal component analysis and B. loadings using sequencing metrics. Colors in A. represent the different tissue sites of origin. C. Ratio of sequence reads aligning to the X and Y chromosome and cutoffs used to infer gender. D. Histogram of average coverage over targeted bases (filtered, aligned reads). Figure S2: VQSR filtering effects on tumor-only mutation detection. A. Fraction of total putative TGS mutations falling in each GATK VQSR tranche (PASS being the most specific, SNPto100 being the least specific). B. Fraction of TGS mutations seen in COSMIC more than five times falling into each VQSR tranche. C. Fraction of total putative WES mutations falling in each GATK VQSR tranche (PASS being most specific, SNPto100 being least specific). D. Fraction of WES mutations seen in COSMIC more than five times falling into each VQSR tranche. Figure S3: Mutation counts after filtering with additional population databases. Boxplots showing numbers of mutations detected after filtering with KAVIAR, ExAC, or both (excluding AF ≥ 1%) in addition to 1000 Genomes and ESP. The rightmost columns show the minimal effect of filtering with KAVIAR and ExAC after the normal filter has been applied. A. TGS cohort, B. WES cohort. Median counts are indicated by the dark line in the middle of the box. The bottom and top of the box are the first and third quartiles, respectively. The whiskers represent the most extreme points within 1.5 times the interquartile range. The y-axes are in the log scale. Figure S4: Normal pool features affect the ability to remove variants. Boxplots showing the putative mutation counts after filtering with titrated sample counts in the normal pool for A. TGS cohort, B. WES cohort. Figure S5: Total nonref counts, precision, and recall with subsequent filters. Total nonref counts (left), precision compare to MuTect (middle), and recall compared to MuTect (right) for A. TGS and B. WES. All plots are in a linear sc [file 40246_2017_118_MOESM3_ESM.pdf]
