# Supplementary material for: Evaluating somatic tumor mutation detection without matched normal samples
Source: Hum Genomics. 2017 Sep 4;11:22. doi: 10.1186/s40246-017-0118-2 (PMC5584341; doi:10.1186/s40246-017-0118-2)
Supplement: Supplementary file 5 — Methods: A detailed description of the analysis methods used for the detection of somatic mutations with an unmatched pool of normal samples. (DOCX 106 kb) [file 40246_2017_118_MOESM5_ESM.docx]

**Supplemental Methods**

**Tumor-only somatic mutation detection, with normal pool**

Settings were initially informed by 1000 Genomes phase 2 and GATK best practices:

<ftp://ftp-trace.ncbi.nih.gov/1000genomes/ftp/README.alignment_data>

https://www.broadinstitute.org/gatk/guide/pdfdocs/GATK_GuideBook_2.3-9.pdf

GATK_Lite 2.2-16 was used - settings may be different for other versions.

Step 0: Raw sequence reads

- Obtain raw sequence reads in the FASTQ file format, preferably GZipped for space.

Step 1: Sequence Alignment

- Create a folder for each sample

- Align with BWA (paired-end):

bwa aln -q 15 <reference>^1^ <FASTQ.1> -f <out.1.sai>

bwa aln -q 15 <reference> <FASTQ.2> -f <out.2.sai>

bwa sampe -a <max_insert_size>^2^ \

-r “@RG\tID:${NAME}\tSM:${NAME}\tPL:ILLUMINA\tLB:${NAME}_lib" \

<reference> \

<out.1.sai> \

<out.2.sai> \

<FASTQ.1> \

<FASTQ.2> \

-f <out.sam>

^1^hs37d5 was used in this study.

^2^A value of 600 was used in this study.

Step 2: SAM to BAM, sort, fixmate, add MD

- Sort, correct with samtools (pre 1.1):

samtools view -bSu <out.sam> | \

samtools sort -n -o -m 3000000000 - <out.sort.tmp> | \

samtools fixmate /dev/stdin /dev/stdout | \

samtools sort -o -m 3000000000 - <out.csort.tmp> | \

samtools fillmd -b - <reference.fa> \

> <out.fixed.bam>

Step 3: Mark duplicates

- Mark duplicates with Picard:

java -Xmx6g –jar MarkDuplicates.jar \

INPUT=<out.fixed.bam> \

OUTPUT=<out.dup.bam> \

ASSUME_SORTED=TRUE \

VALIDATION_STRINGENCY=LENIENT \

METRICS_FILE=<out.dup.metrics> \

CREATE_INDEX=TRUE

Step 4: Realign around indels

- low_coverage and mills_devine indel VCFs from GATK bundle

- Indel Realignment with GATK:

java -Xmx6g -jar GenomeAnalysisTK.jar \

-T RealignerTargetCreator \

-R <reference.fa> \

-I <out.dup.bam> \

-o <out.intervals> \

-known <low_coverage_indels.vcf> \

-known <mills_devine_indels.vcf> \

java -Xmx6g -jar GenomeAnalysisTK.jar \

-T IndelRealigner \

-R <reference.fa> \

-I <out.dup.bam> \

-targetIntervals <out.intervals> \

-o <out.realign.bam> \

-known <low_coverage_indels.vcf> \

-known <mills_devine_indels.vcf> \

-LOD 4.0 \

-model USE_READS

Step 5: Base quality recalibration

- dbsnp.vcf from GATK bundle

- BQSR with GATK:

java -Xmx6g -jar GenomeAnalysisTK.jar \

-T CountCovariates \

-l INFO \

-L ‘<chr1;chr2;...>’^3^ \

-R <reference.fa> \

-I <out.realign.bam> \

-knownSites <dbsnp.vcf> \

-cov ReadGroupCovariate \

-cov QualityScoreCovariate \

-cov CycleCovariate \

-DinucCovariate \

-recalFile <out.recal_data>

^3^If parallelizing, identify the appropriate region here.

java -Xmx6g -jar GenomeAnalysisTK.jar \

-T TableRecalibration \

-l INFO \

-R <reference.fa> \

-I <out.realign.bam> \

-o <out.recal.bam> \

-recalFile <out.recal_data>

Step 6: Add MD tag and index final BAM

- Add MD tag with samtools:

samtools calmd -Erb <out.recal.bam> <reference.fa> \

> <out.bam>

samtools index <out.bam>

Step 7: Collect metrics

- Get alignment metrics with Picard

java -Xmx6g -jar CollectMultipleMetrics.jar \

INPUT=<out.bam> \

REFERENCE_SEQUENCE=<reference.fa> \

OUTPUT=<out.stats> \

VALIDATION_STATUS=LENIENT

Step 8: Clean temporary files for each sample alignment directory

- remove intermediate BAM, SAM, and other files

Step 9: Multi-sample genotyping

- multi-sample genotyping with GATK UnifiedGenotyper; get genotypes for ALL

samples at variant positions, even when reference

- all samples together (tumor and unmatched normal)

- may need to merge groups of bamfiles together (to avoid exceeding number of open

files)

- split by chromosome for parallelization,

but may need to further split chromosomes into subregions

# merge bams if needed with Picard

java -Xmx6g -jar MergeSamFiles.jar \

OUTPUT=<merge_n.bam> \

ASSUME_SORTED=true \

VALIDATION_STRINGENCY=LENIENT \

INPUT=<out.1.bam> \

INPUT=<out.2.bam> \

...

INPUT=<out.n.bam>

samtools index merge_n.bam

java -Xmx40g -jar GenomeAnalysisTK.jar \

-T UnifiedGenotyper \

-R <reference.fa> \

-o <out.vcf> \

-D <dbsnp_sites.vcf>^4^ \

-glm BOTH \

-out_mode EMIT_VARIANTS_ONLY \

-A DepthOfCoverage \

-A AlleleBalance \

-A FisherStrand \

-L <interval>^5^ \

-I <list_of_bamfiles>

^4^dbSNP135 from the 1000Genomes PhaseII mapping was used in this study: “ALL.wgs.dbsnp.build135.snps.sites.vcf.gz”

^5^If parallelizing, identify the appropriate region here.

# Then, concatenate all interval files in order, BGZip, and index with tabix.

Step 10: Filter against target file (plus flanking bases)

- use BedTools to only retain positions in or near targeted regions

intersectBed -header -u \

-a <out.vcf> \

-b <target_uniq_padded.bed> \

| bgzip -c > <out.target.vcf.gz>

tabix -p vcf <out.target.vcf.gz>

Step 11: Variant quality score recalibration

- COSMIC training set downloaded as VCF from COSMIC, filtered to include sites with

count >= 1.

- other training files from GATK bundle

- these settings may need tweaking for different target sizes

- VQSR with GATK using cancer-specific settings, COSMIC training set:

java -Xmx25g -jar GenomeAnalysisTK.jar \

-T VariantRecalibrator \

-R <reference.fa> \

-input <out.target.vcf.gz> \

-resource:hapmap,VCF,known=false,training=true,truth=true,prior=15.0 snp.vcf \

-resource:omni,VCF,known=false,training=true,truth=false,prior=12.0 omni.vcf \

-resource:dbsnp,VCF,known=true,training=false,truth=false,prior=6.0 dbsnp.vcf \

-resource:cosmic,VCF,known=true,training=true,truth=true,prior=12.0 cosmic.vcf \

-an MQRankSum -an ReadPosRankSum -an FS -an MQ \

-recalFile <out.snv.recalfile> \

-tranchesFile <out.snv.output.tranches> \

-rscriptFile <out.snv.output.plots> \

-mode SNP \

--maxGaussians 4

java -Xmx25g -jar GenomeAnalysisTK.jar \

-T VariantRecalibrator \

-R <reference.fa> \

-input <out.target.vcf.gz> \

-resource:mills,VCF,known=true,training=true,truth=true,prior=12.0 mills_indel.vcf \

-an ReadPosRankSum -an FS \

-recalFile <out.div.recalfile> \

-tranchesFile <out.div.output.tranches> \

-rscriptFile <out.div.output.plots> \

-mode INDEL \

-std 10.0 \

--percentBadVariants 0.12 \

--maxGaussians 4

java -Xmx25g -jar GenomeAnalysisTK.jar \

-T ApplyRecalibration \

-R <reference.fa> \

-input <out.target.vcf.gz> \

--ts_filter_level 99.90 \

-tranchesFile <out.snv.output.tranches> \

-recalFile <out.snv.recalfile> \

-mode SNP \

-o <out.snv.recal.vcf>

java -Xmx25g -jar GenomeAnalysisTK.jar \

-T ApplyRecalibration \

-R <reference.fa> \

-input <out.snv.recal.vcf> \

--ts_filter_level 99.0 \

-tranchesFile <out.div.output.tranches> \

-recalFile <out.div.recalfile> \

-mode INDEL \

-o <out.recal.vcf>

bgzip <out.recal.vcf>

tabix -p vcf <out.recal.vcf.gz>

Step 12: Subset the normal samples, recalculate AF, AN, AC

- custom Perl scripts to extract normal samples, recalculate population metrics on just

these samples

Step 13: Subset unique tumor samples, recalculate AF, AN, AC

- custom Perl scripts to extract tumor samples, recalculate population metrics on just

these samples

Step 14: Annotate tumor VCF with normal VCF frequencies

- use custom Perl scripts to extract population metrics (AF) from the normal file, insert

into the tumor file at the exact position/alternate base location

# Can now either hard filter out positions where normal freq exceeds desired threshold, or do it later during analyses.

# In this study, we excluded any variants observed in the described population databases (1000 Genomes, NHLBI ESP). To avoid the chance of exclusion of rare disease associated variants, users may prefer to set a threshold of 1% or even 5%.

Step 15: Annotate VCF as desired

- Annotate against gene models using ANNOVAR, snpEFF, VEP, etc.

- use custom Perl scripts to insert COSMIC, 1000 Genomes, other information

Step 16: Downstream analysis

- custom analysis scripts

- if smaller sample size, visualize with tools like VarSifter
